# A Software Defined Radio Evaluation Platform for WBAN Systems

**DOI:** 10.3390/s18124494

**Published:** 2018-12-19

**Authors:** Junchao Wang, Kaining Han, Zhiyu Chen, Anastasios Alexandridis, Zeljko Zilic, Yu Pang, Jinzhao Lin

**Affiliations:** 1 Department of Electrical and Computer Engineering, McGill University, Montreal, QC H3A 2A7, Canada; junchao.wang@mail.mcgill.ca (J.W.); zhiyu.chen3@mail.mcgill.ca (Z.C.); anastasios.alexandridis@mail.mcgill.ca (A.A.); zeljko.zilic@mcgill.ca (Z.Z.); 2 National Key Laboratory of Science and Technology on Communications, University of Electronic Science and Technology of China, Chengdu 611731, China; 3 Chongqing University of Posts and Telecommunications, Chongqing 400065, China; pangyu@cqupt.edu.cn (Y.P.); linjz@cqupt.edu.cn (J.L.)

**Keywords:** software-defined radio, wireless body area network, evaluation platform

## Abstract

In recent years, the Wireless Body Area Network (WBAN) concept has attracted significant academic and industrial attention. WBAN specifies a network dedicated to collecting personal biomedical data from advanced sensors that are then used for health and lifestyle purposes. In 2012, the 802.15.6 WBAN standard was released by the Institute of Electrical and Electronics Engineers (IEEE), which regulates and specifies the configurations of WBAN. Compared to the prevailing wireless communication technologies such as Bluetooth and ZigBee, the WBAN standard has the advantages of ultra-low power consumption, high reliability, and high-security protection while transmitting sensitive personal data. Based on the standard specification, several implementations have been published. However, in terms of evaluation, different designs were implemented in proprietary evaluation environments, which may lead to unfair comparison. In this paper, a Software-Defined Radio (SDR) evaluation platform for WBAN systems is proposed to evaluate the RF channel specified in the IEEE 802.15.6 standard. A narrowband communication protocol demonstration with a security scheme in WBAN has been performed to successfully validate the design in the proposed evaluation platform.

## 1. Introduction

**Figure 1:**
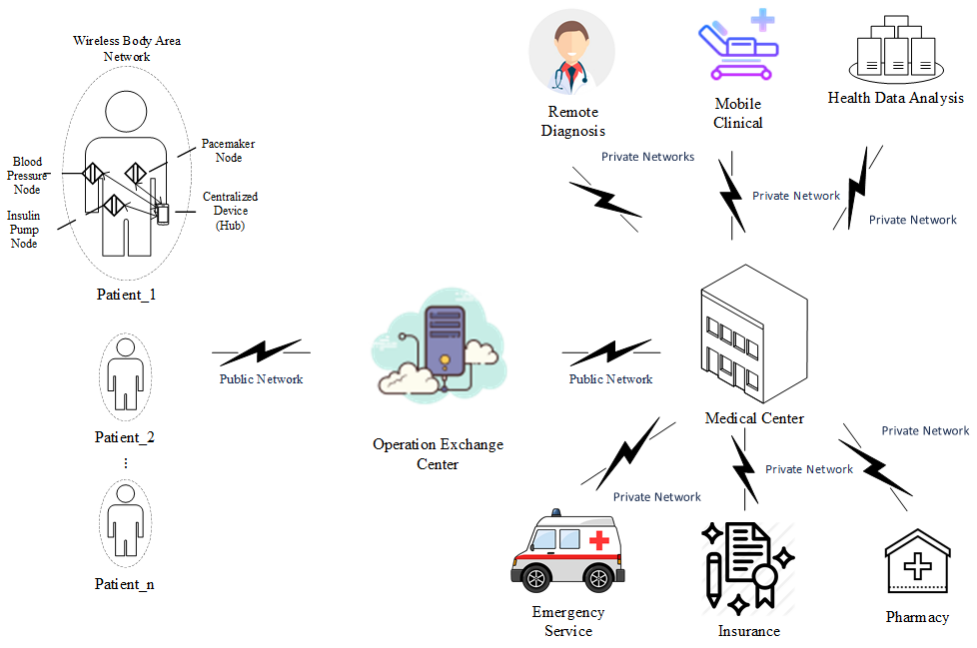
Architecture of an intelligent healthcare system.

Based on the data provided by the Canadian Institution for Health Information (CIHI), the average health expenditure for every individual in Canada was 6604 Canadian dollars in 2017, which requires 11.5% of the overall Gross Domestic Product (GDP), up from only 7% of the GDP in 1975. In other words, Canadians spent 4.5% more of their wealth on healthcare over the past 43 years [1]. Meanwhile, according to the report provided by Bacchus Barua from the Fraser Institute [2], the average waiting time for consulting medical professionals was 21.2 weeks in 2017 in Canada due to the shortage of medical professionals, even though they have spent a huge amount of their income on healthcare. Therefore, there is a strong demand for an economical and efficient healthcare solution, which is capable of also addressing the shortage of medical professionals. One such solution can be a secured intelligent healthcare system, which can not only monitor the physical conditions of the patients remotely, but also analyze the potential physical issues the patients are facing and provide feedback to them, as demonstrated in Figure 1.

By benefiting from the rapid development of modern technology, increasing types of biomedical data can be collected from patients and transmitted to the cloud for further data processing and storage. This is especially true with the rapid growth in advanced biomedical sensors, such as Electroencephalogram (EEG) and Electrocardiography (ECG) sensors [3] and blood pressure sensors [4], as well as wireless networking, such as the Fifth Generation (5G) cellular mobile standards and Bluetooth Low Energy (BLE). However, there are two potential issues that still restrict the development of intelligent healthcare systems [5,6]. First, the sensors on humans are extremely power-sensitive, especially the implanted sensors with limited power supply and the inconvenience of battery change. The most commonly-utilized wireless communication technologies in the proposed sensors are Bluetooth and ZigBee, which are not dedicated and optimized for biomedical data transmission. Secondly, the data collected from patients are private and critical, which could cause serious problems if the information were tampered with. Hence, an efficient and unique security scheme is also necessary for wireless communications in the intelligent healthcare system.

**Figure 2:**
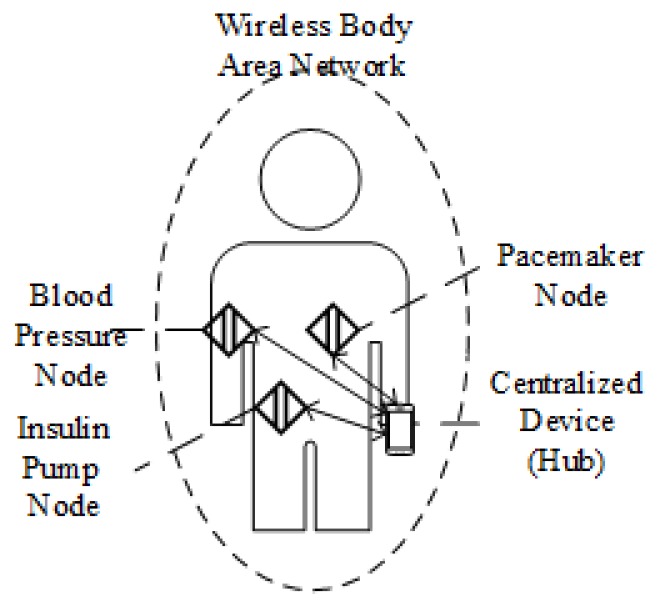
Architecture of a typical WBAN.

WBANs, illustrated in Figure 2, have attracted huge academic and industrial attention in recent years because they define the shared communication infrastructure for wireless data transmission between sensors and other devices. Starting in 2012, the Institute of Electrical and Electronics Engineers (IEEE) released the 802.15.6 standard, which specifies and regulates the detailed configurations of WBANs. Based on the specifications of the IEEE 802.15.6 standard, multiple hardware-based and software-based implementations of WBAN have been proposed [7,8,9]. The evaluation results of the implementations illustrate that WBAN has advantages in power consumption, privacy protection, and efficient communication for biomedical data. For instance, the power consumption of WBAN systems is between 0.1 mW–5 mW approximately when the transmitting data rate is 1 Mbps, while for the same transmitting data rate, Bluetooth consumes between 5 mW and 100 mW approximately. In this case, the battery can last approximately one year in WBAN systems, while it can only last less than a month in Bluetooth systems [5], as illustrated in Table 1.

However, since the IEEE 802.15.6 standard supports three types of communications (Narrowband (NB), Ultra Wideband (UWB), and Human Body Communication (HBC)) and each communication corresponds to various transmission specifications such as encoding methods, modulations, and transmission frequencies, the designs were implemented in different platforms for evaluation purposes. However, evaluating different implementations of WBAN in various platforms could cause certain issues. On the one hand, the evaluation results are affected by different configurations of the platforms, such as the performance of the Field-Programmable Gate Array (FPGA), Random-Access Memory (RAM), and Read-Only Memory (ROM), which leads to unfair comparisons among different designs. On the other hand, establishing evaluation platforms for every individual implementation of WBAN is not only time consuming for the researchers and engineers, but it also increases the complexity of the design. Since each RF front-end design and fabrication can take many months, a platform is needed that can drastically speed up the evaluations.

The motivation of this research is to provide a rapidly-configurable SDR evaluation platform for WBAN systems, which not only can provide test cases that can help evaluate different modules in different environments (e.g., the Bit Error Rate (BER) for different distances), but also be reproducible in multiple implementation platforms. To provide a wider benefit to the research community, all the source code for this SDR platform is posted to a publicly-accessible GitHub repository. The SDR evaluation platform may be used to evaluate and prototype different applications, including, but not limited to, healthcare networks and vehicular networks [10], which were found to be good candidates for WBAN implementations.

Further, to ensure that the proposed SDR evaluation platform can be supported on various FPGA platforms (other than the MiniBEE), the verification has been performed in the Xilinx Kintex-7 FPGA KC705 Evaluation Kit (Xilinx, Inc., San Jose, CA, USA) and the Altera Arria 10 SoC Development Kit (Intel, Santa Clara, CA, USA).

Our contributions are as follows. Firstly, the procedure of designing and validating the WBAN systems is dramatically shortened by utilizing the proposed evaluation platform, since there is no need to build circuits for each specific WBAN system, especially when the transceivers take exorbitantly long time to be developed in Application-Specific Integrated Circuits (ASICs).

Secondly, it is more feasible for researchers to evaluate the real performance of a certain optimized module in the WBAN by simply replacing the module in the evaluation platform and comparing the performance. By selecting the appropriate hardware on which the evaluation platform can be implemented, which would be dependent on the application, and evaluating two different modules on it, module optimization can be carried out to a certain extent. At the same time, the evaluation platform can be implemented in a different hardware, should a different application require that. Thirdly, it provides a fair comparison platform to evaluate different designs for WBAN systems at the RTL fferent circuit synthesis technologies.

The rest of this paper is organized as follows. Section 2 provides the background and previous work focusing on the IEEE 802.15.6 standard and the general specifications of the SDR testbed. Functionality, structure, and hardware components of the proposed evaluation platform for WBAN systems are detailed in Section 3. The implementation of the proposed testbed for WBAN and a demo performance of a baseband processing module with a WBAN security scheme is shown in Section 4. Section 5 concludes the paper and provides the future work guide.

## 2. Preliminary and Related Work

**Figure 3:**
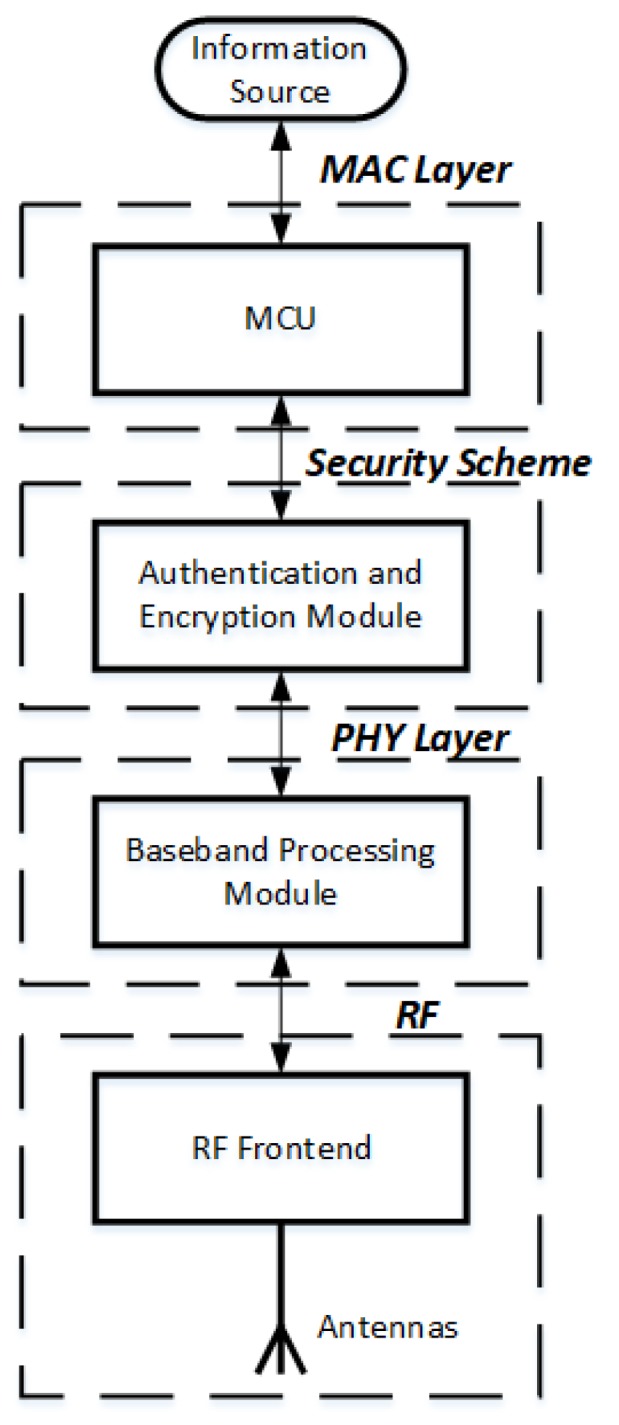
Transmission flow of a WBAN that has been specified in the IEEE 802.15.6 standard.

The IEEE 802.15.6 standard specifies three types of communications: NB, UWB, and HBC. Each communication type defines various configurations for the network. However, the processing flow is similar for different communications in WBAN. As specified in the IEEE 802.15.6 wireless body area network standard [11], the transmission flow of WBAN is mainly separated into four parts, which are the Medium Access Control (MAC) layer, security scheme, and Physical (PHY) layer, as shown in Figure 3. Initially, the MAC layer specifies the MAC frame format and the communication modes in the network [12], which requires a Microcontroller Unit (MCU) to process. Afterward, the security scheme needs to determine whether the link needs to be authenticated and encrypted, based on the security level of communication. The PHY layer involves the baseband processing module, where it processes the original binary data from the security scheme into a format that is suitable for processing in the RF front-end, where it is transmitted. Precisely, the responsibility of the baseband processing module is the activation and deactivation of the radio transceiver, clear communication assessment, and data reception and transmission [12]. Last but not least, the RF front-end converts the digital data into an analog signal modulated at the right frequency, passes the modulated signal to an amplifier, and transmits it by the antenna (vice versa for the receiver).

### 2.1. Specifications of IEEE 802.15.6

#### 2.1.1. MAC Layer of WBAN

**Figure 4:**
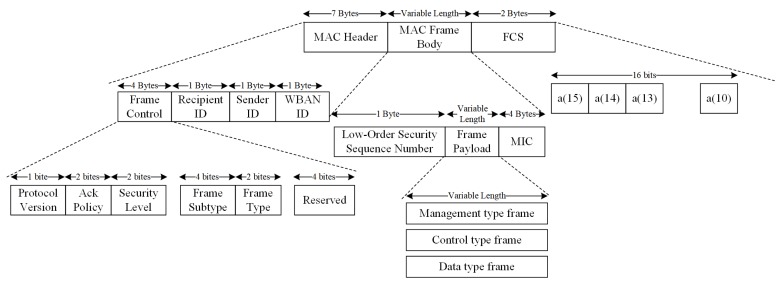
The frame structure of a general MAC frame defined in IEEE 802.15.6. FCS, Frame Check Sequence.

Sitting above the PHY Layer, the MAC layer of IEEE 802.15.6 is designed to control communication access. To do this, the MAC layer divides the entire communication into a chain of superframes through the hub (coordinator). At the boundary of these superframes, the hub chooses beacon periods of equal length to bound each superframe. If needed, one could shift the offset of the beacon period through the hub. The superframes will be normally sent in each beacon period [5], unless there is a restriction by regulations in the Medical Device Radiocommunications Service (MICS) band or the superframes are inactive. Figure 4 provides an overview of the structure of superframes in the standard. The superframe can be divided into three different components, the MAC header, the MAC frame body, and the Frame Check Sequence (FCS). With a length of seven bytes, the MAC header can be further divided into recipient ID, sender ID, WBAN ID, and frame control, which contains information such as protocol version, ack policy, and so on. The MAC frame body has a variable length; it contains low-order security sequence number, frame payload with selected types, and MIC. The last two bytes of a MAC frame are the FCS to detect possible errors in transmission. The standard specifies the CRC-16-CCITT sequence to be used in error detection. The Cyclic Redundancy Check (CRC) polynomial is shown in Equation (1), where $a_{15}$ is the Least Significant Bit (LSB) of the field, $a_{0}$ is the Most Significant Bit (MSB), and $a_{15}$, $a_{14}$, …, $a_{0}$ are the binary coefficients.

(1) $$G\left(x\right)=x^{16}+x^{12}+x^{5}+1$$

#### 2.1.2. Security Scheme of WBAN

The IEEE 802.15.6 standard specifies three security levels for all communications in WBAN, Level 0, Level 1, and Level 2, respectively. Level 0 is unsecured communications, where neither authentication nor encryption are required. Public information, such as time stamps, which is neither critical nor private, could be transmitted at this security level. Meanwhile, the communication of security Level 1 contains private, but not critical data, such as names, ages, and locations. These data are not significant for the physical conditions of the patients; however, they would still not want to release it to the public. In these cases, authentication is required, while encryption is not involved. In the case of the most critical data, such as blood pressure, heart rate, and parameters for a pacemaker, it is a requirement for them to be transmitted at security Level 2. Both authentication and encryption are mandatory for Level 2 communication.

In terms of the methods implementing authentication and encryption, the standard specifies the certificate validation as the authentication method and Elliptic-Curve Cryptography (ECC) as the encryption method. Based on the specifications, multiple security schemes have been proposed to implement and even improve the security protection of WBAN systems. Precisely, lightweight data authentication schemes have been proposed in [13,14], which achieved much lower power consumption than conventional WBAN security schemes. Moreover, the work in [15,16] proposed data authentication and encryption methods by utilizing the data collected from the sensors to generate dynamic keys, which simplifies the system complexity of the security scheme while increasing the security level. Furthermore, an ASIC implementation of security scheme for WBAN has been proposed [8]. In this design, besides validating the certificate, there is a second phase for the authentication procedure called the challenge-response phase to increase the security protection of the communications in WBAN.

#### 2.1.3. PHY Layer of WBAN

As previously mentioned, in the specifications of the IEEE 802.15.6-2012 standard, there are three communications that could be utilized in the communication of WBAN: NB, UWB, and HBC, respectively. As a typical case, the physical layer processing of NB communication is analyzed as follows.

**Figure 5:**
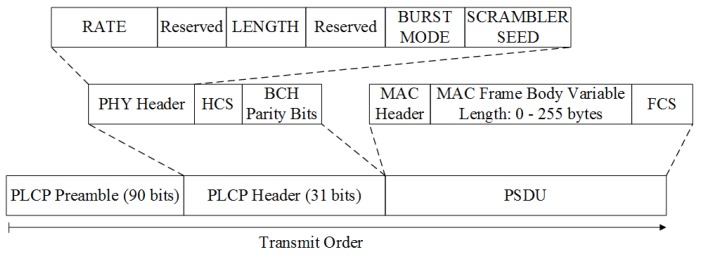
Standard Physical-Layer Protocol Data Unit (PPDU) structure. PLCP, Physical-Layer Convergence Protocol; PSDU, Physical-Layer Service Data Unit; HCS, Header Check Sequence.

The standard Physical-Layer Protocol Data Unit (PPDU) for NB is illustrated in Figure 5. Every PPDU contains three main components: the Physical-Layer Convergence Protocol (PLCP) preamble, the PLCP header, and the Physical-Layer Service Data Unit (PSDU).

• PLCP Preamble

As seen in Figure 5, the 90-bit PLCP preamble is the initial data package that needs to be sent for every PPDU to assist the receiver in data packet detection, timing synchronization, and carrier recovery. The data packet detection, timing synchronization, and the carrier recovery are presented next.

• PLCP header

Following the PLCP preamble, a 31-bit PLCP header that contains a 15-bit PHY header, a 4-bit Header Check Sequence (HCS), and 12-bit Bose Chaudhuri Hocquenghem (BCH) parity bits, can be found. The BCH code with 19 information bits and 12 parity check bits (31,19,2) is a shorter version of standard BCH code (63,51,2), which provides up to *t* = 2 error bit correction capability. The purpose of the PLCP header is to provide the system configuration parameters related to the receiver.

The PHY header is constructed by a 3-bit rage, 8-bit length, 1-bit burst mode, and 1-bit scrambler seed, while two bits are reserved. The detailed encoding methods and corresponding meaning of them are specified in the IEEE 802.15.6 standard.

• PSDU

The PSDU is the component that contains the data from the Medium Access Control (MAC) layer. More precisely, it consists of a 7-byte MAC header at the beginning of the sequence, a 2-byte FCS at the end of the sequence, and a 0–255-byte MAC frame body in the middle containing the data. In this paper, the MAC frame body length is fixed to 255 bytes to simplify the control logic of the transmitter.

**Figure 6:**
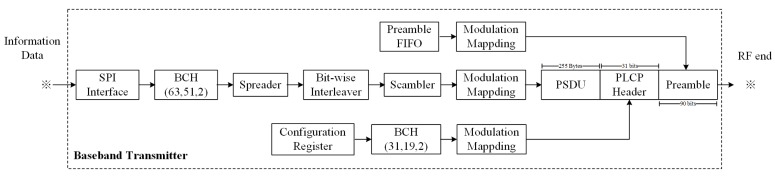
The block diagram of the baseband transmitter.

The block diagram of the baseband transmitter is illustrated in Figure 6. According to the IEEE 802.15.6 standard, the modulation for the preamble, PLCP header, and PSDU is $\frac{\pi }{2}$-Differential Binary Phase Shift Keying (DBPSK), $\frac{\pi }{2}$-DBPSK, and $\frac{\pi }{4}$-Differential Quadrature Phase Shift Keying (DQPSK), respectively.

### 2.2. Radio Frequency Characteristic of WBAN

The IEEE 802.15.6 standard covers a series of operating frequency bands: 402 MHz–405 MHz, 420 MHz–450 MHz, 863 MHz–870 MHz, 902 MHz–928 MHz, 950 MHz–958 MHz, 2360 MHz–2400 MHz, and 2400 MHz–2483.5 MHz. Each frequency band includes several sub-channels, which are shown in Table 2, where $g_{1}\left(n_{c}\right)$ and $g_{2}\left(n_{c}\right)$ are mapping functions used in the 420 MHz–450 MHz and 863 MHz–870 MHz frequency bands, respectively.

(2) $$g_{1}\left(n_{c}\right)=\left\{\begin{array}{cc} n_{c}, & 0\leq n_{c}\leq 1;\\ n_{c}+6.875, & 2\leq n_{c}\leq 1;\\ n_{c}+13.40, & n_{c}=5;\\ n_{c}+35.025, & 6\leq n_{c}\leq 7;\\ n_{c}+40.925, & 8\leq n_{c}\leq 9;\\ n_{c}+47.250, & 10\leq n_{c}\leq 11; \end{array}\right.$$

(3) $$g_{2}\left(n_{c}\right)=\left\{\begin{array}{cc} n_{c}, & 0\leq n_{c}\leq 7;\\ n_{c}+0.5, & n_{c}=8;\\ n_{c}+1, & 9\leq n_{c}\leq 12;\\ n_{c}+1.5, & n_{c}=13; \end{array}\right.$$

Based on the different bandwidth of these frequency bands and using the corresponding baseband parameter configuration, the data transmission rate is shown in Table 3.

### 2.3. SDR Testbed

The traditional industrial process of developing a digital communication system is to use an ASIC to perform the RF test. However, despite having the advantages of great performance, low power, and reduced footprint, using an ASIC, on the other hand, raises a high non-recurring cost and dramatically increases the development time. Unlike the ASIC implementation, SDR testbeds provide users with great flexibility to evaluate the RF performance of a certain design. For a common SDR system, the data processing task is performed by a Digital Signal Processor (DSP) or by an FPGA. Due to the programmable nature of the DSP and FPGA, users working with SDR platforms have the ability to change the parameters of the system based on their requirements [17]. In industry, SDR testbeds have been widely adopted as verification tools in wireless communications by the researchers working with other technologies such as Long-Term Evolution (LTE) [18], WLAN [19], Bluetooth [20], and 5G technologies [21]. For instance, in the article [22], the authors proposed a new design architecture to provide effective spectrum management for 5G wireless networks by applying it on an SDR platform. On the contrary, there is still no existing SDR evaluation platform for the latest IEEE 802.15.6 standard. Meanwhile, the IEEE 802.15.6 standard defined a wide range of frequency channels and multiple modulation methods for applications under different circumstances [12]. This highly adaptive nature raises the need for an equally flexible evaluation platform. Due to the configurable nature of the SDR platform, switching between different frequency channels and modulation methods can be achieved easily, which was earlier impossible without manufacturing multiple ICs that implement different configurations.

## 3. Proposed SDR Evaluation Platform for WBAN Systems

### 3.1. Functionality Description

As mentioned in Section 2, WBAN supports three types of communications, namely NB, UWB, and HBC channel. Various methods of encoding, operation frequency, modulation, and other communication parameters shall be utilized in different communications in WBAN systems. Further, the security scheme could also vary based on the security level of the communications. Therefore, for the evaluation purpose of WBAN systems, all supported configurations including methods of encoding and decoding, operation frequency, methods of modulation, and specific security scheme need to be implemented in the evaluation platform. In this paper, an SDR evaluation platform for WBAN systems is proposed that supports all the communications specified in the IEEE 802.15.6 standard utilizing RF as a carrier. In addition, since HBC does not utilize RF as the communication carrier, it is not supported in the proposed evaluation platform. The detailed architecture and specifications are given in the subsections to follow.

### 3.2. Hardware Architecture

**Figure 7:**
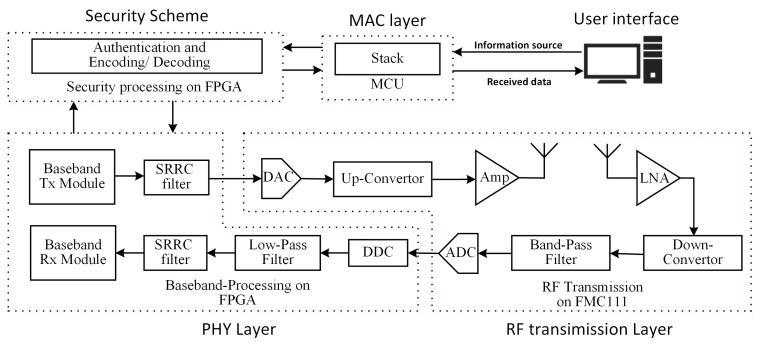
Architecture overview of the proposed Software-Defined Radio (SDR) platform. SRRC, Square Root Raised Cosine; DDC, Digital Down-Converter. LNA, Low-Noise Amplifier.

In the proposed SDR evaluation platform, the information source generated from the user interface can be transmitted through three different hardware components in a designated sequence. As illustrated in Figure 7, after the information is sent from the user interface, it will be first transmitted into the protocol stack for frame formation implemented in the MCU. Then, these frames will be authenticated and encoded following the given security scheme of the IEEE 802.15.6 standard on an FPGA. Once the encoding process is done, the encrypted data go into the baseband processing step implemented in an FPGA. Here, the encrypted message will be modulated and filtered through a Square Root Raised Cosine (SRRC) filter under the specified settings. After that, this signal will be passed into an FPGA Mezzanine Cards (FMC) 111 RF board. In this RF module, the digital signal will first be converted into an analog signal and will then be stepped up to the radio frequency for transmission through the antenna. On the receiver side, once the antenna receives the transmitted signal and the signal passes the Low-Noise Amplifier (LNA), it will downconvert the RF signal to a Medium Frequency (MF) of 30.72 MHz to meet the relatively low sampling rate of the Analog to Digital Converter (ADC). After getting the digital signal, the signal will be passed into the Digital Down-Converter (DDC) to shift the MF down to the baseband. Once the baseband signal is obtained, it is passed through a low-pass filter to filter out the harmonic frequencies. The remaining processes are just the inversion of the previous processes. Once the signal is passed through the SRRC filter, which is compatible with the sending end SRRC, it is demodulated in the baseband receiver module. Then, the security scheme applied to FPGA decodes the data and sends it to the protocol stack for de-framing. Finally, the extracted data are sent back to the user interface.

**Figure 8:**
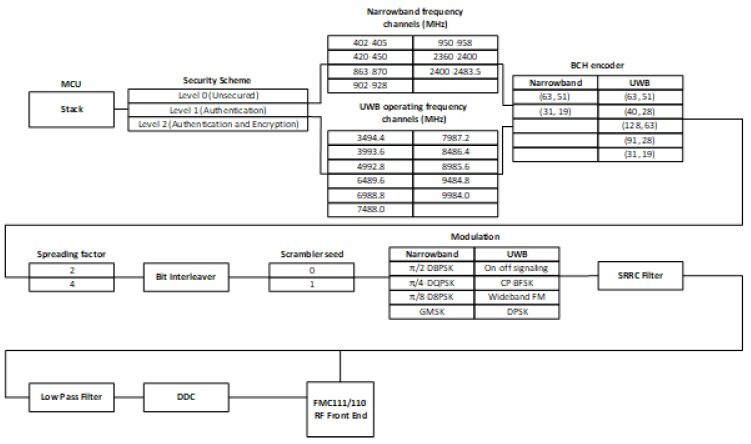
Implementation architecture of the proposed SDR platform.

The detailed configurations of the proposed evaluation platform are demonstrated in Figure 8. As can be seen from the figure, every individual block is reconfigurable as needed. Once the configuration of a certain block is determined, the corresponding modules will be activated, while other modules that are not utilized will be disabled to reduce the hardware cost of the platform.

For example, assume that the security level of a certain communication has been determined to be Level 1, which requires authentication, but not encryption. In this case, the authentication module of the security scheme in the evaluation platform is activated, while the encryption module is disabled. Based on the types of communications, different operating frequencies are distributed. For NB communication, the proposed evaluation platform supports seven RF bands from 400 MHz–2.4 GHz, while it also supports 11 RF bands from 3494.4 MHz–9984.0 MHz for the UWB communications. Meanwhile, even though both NB and UWB utilize BCH as the coding method for the communication, there are still various configurations for them. The proposed platform supports all the configurations for BCH encoding and decoding required by the standard, as demonstrated in the figure. Moreover, there are eight methods of modulation that can be configured in the platform: $\frac{\pi }{2}$-DBPSK, $\frac{\pi }{2}$-DBPSK, $\frac{\pi }{4}$-DQPSK, and GMSK for NB communications and on-off signaling, CP-BFSK, wideband FM, and DPSK for UWB communications. The proposed evaluation platform shares the blocks for spreading factor, bit interleaver, scrambler seed, SRRC filter, low-pass filter, and DDC for both NB and UWB communications, since they have identical configurations.

## 4. Implementation and Demonstration

### 4.1. Implementation Architecture of Proposed Evaluation Platform for WBAN Systems

**Figure 9:**
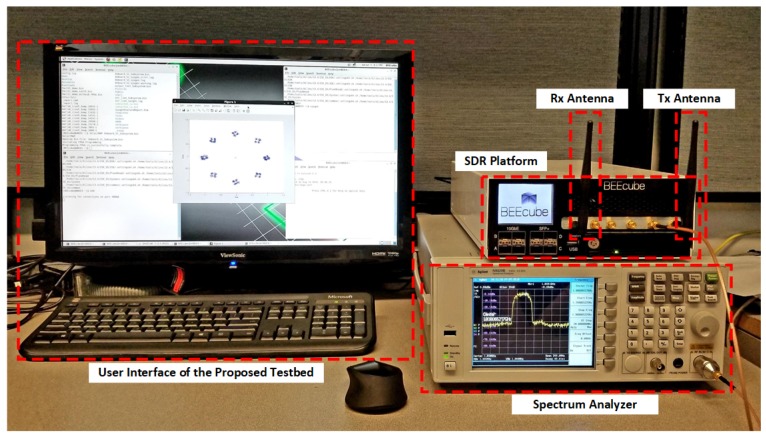
Demonstration of the proposed SDR evaluation platform for WBAN systems.

The proposed SDR evaluation platform is implemented on a MiniBEE4 platform provided by Canadian Microelectronics Cooperation (CMC), Kingston, ON, Canada. The MiniBEE4 contains a Xilinx Virtex-6-XC6VSX475T FPGA, San Jose, CA, USA connected with a configurable FMC111/110 RF front-end and a personal computer with CentOS running in it. In addition, two isotropic antennas are attached to the RF front-end. At the same time, an Agilent Infiniium DSA80000B spectrum analyzer (Agilent, Santa Clara, CA, USA) is utilized to determine the frequency characteristics and verify the performance of the RF front-end, as shown in Figure 9.

The User Interface (UI), connected with the MiniBEE4 SDR Platform, identifies the information source and received data, while the stack (MAC layer) is running on the CPU of the Personal Computer (PC). The security scheme that contains an authentication module and encryption module is implemented in the FPGA, while the reconfigurable baseband processing module of the Physical layer (PHY) is also performed in the integrated Xilinx Virtex-6-XC6VSX475T FPGA (Xilinx, Inc., San Jose, CA, USA).

In terms of the configurations of the RF front-end found in the physical layer, a typical configuration of the RF channel is defined as shown in Table 4. Since the MiniBEE4 platform integrates a reconfigurable FMC111/110 RF front-end, all the required operation frequencies specified in the IEEE 802.15.6 standard are supported and can be reconfigured through the RF setup.

### 4.2. Demonstration of Evaluating a Baseband Processing Module with a Security Scheme for WBAN Performed in the Proposed Design

To further evaluate and verify the functionality of the proposed SDR evaluation platform for WBAN systems, a baseband processing module [7] with a security scheme [8] designed for WBAN has been implemented and evaluated in the proposed evaluation platform. Validating the RF channel functionality in various scenarios was the primary interest.

**Figure 10:**
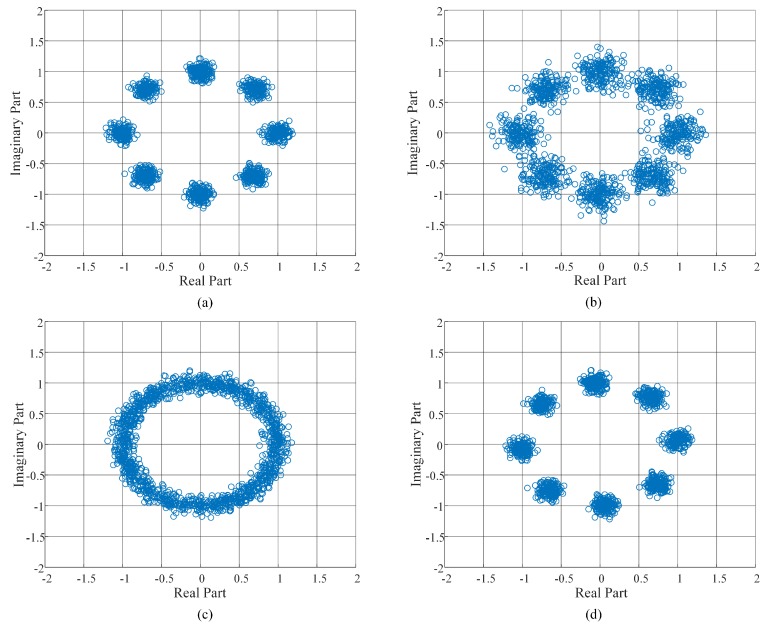
Constellation maps performed in the proposed evaluation platform for [7] ((**a**) Short distance; (**b**) long distance; (**c**) frequency-offset (max. 20 ppm in IEEE 802.15.6); (**d**) corrected frequency-offset).

**Figure 11:**
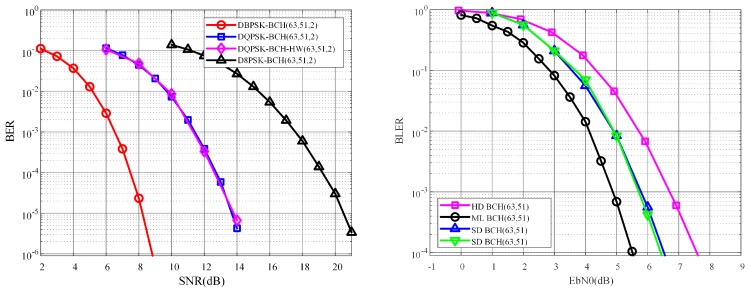
Communication performance executing in the proposed SDR evaluation platform for WBAN systems.

In terms of the demonstration, to assess the modulation scheme, Figure 10 demonstrates the constellation map for four cases. To be more precise, initially, there is a short distance between the two antennas (1 m), and the constellation map from the receiver is shown as Figure 10a. It can be observed that the transmission quality could be guaranteed at a one-meter distance. Afterward, a longer distance between two antennas (2 m) was applied, and Figure 10b illustrates the constellation map for that scenario. In this case, even though the transmission quality seems not as good as that in Figure 10a, the bit error rate can be further improved by the BCH decoding methods. Moreover, in the circumstance of Figure 10c, a practical transmission link, where the maximum frequency offset between the transmitter and the receiver is specified in the standard (20 ppm), is considered. Last but not least, after the frequency offset correction, the constellation map is demonstrated in Figure 10d, which shows satisfying transmission performance. The overall transmission performance is expressed by the Bit Error Rate (BER) vs. Signal to Noise Ratio (SNR) for different modulation methods, as shown in the left side of Figure 11. In the left figure, the pink line which is mostly overlapped with the blue line is the hardware (HW) performance result running in the proposed evaluation platform. It illustrates that the HW performance matches the simulation results.

In order to improve the performance of the communication in WBAN systems, multiple BCH decoding methods are applied to replace the original hard-decision (HD) decoder specified in the IEEE 802.15.6 standard. Therefore, HD decoding, soft-decision (SD) decoding, and maximum-likelihood (ML) decoding methods for BCH (63,51) configurations have been simulated as demonstrated on the right side of Figure 11. The blue line is the performance results, Block Error Rate (BLER) vs. $E_{b}/N_{0}$, running in the proposed evaluation platform for the soft-decoding method, which meets the simulation results.

Furthermore, Table 5 demonstrates the hardware cost of implementing the baseband processing module in the proposed evaluation platform for WBAN systems. Moreover, the security scheme proposed in [8] has been utilized and implemented in the evaluation platform as the authentication and encryption module for the demo communication. The communication level was set to Level 2, which requires both authentication and encryption. Hardware utilization of the security scheme performing in the proposed evaluation platform is shown in Table 6. It could be found that the FPGA platform hardware resource utilization was quite low, which means the SDR platform can support more complicated functional tests and validations at the same time.

### 4.3. Discussion

For NB communications, the proposed evaluation platform supports seven RF bands from 400 MHz–2.4 GHz. However, for the UWB communications, 11 RF bands from 3494.4 MHz–9984.0 MHz with a 499.2 MHz bandwidth, as shown in Table 7, can not be covered by the FMC111 RF module. Meanwhile, both NB and UWB utilize BCH as the coding method for the communications. The proposed platform supports all the configurations for BCH encoding and decoding required by the standard. Moreover, there are eight methods of modulation that can be configured in the platform: $\frac{\pi }{2}$-DBPSK, $\frac{\pi }{2}$-DBPSK, $\frac{\pi }{4}$-DQPSK, and GMSK for NB communications and on-off signaling, Continuous Phase Fre-quency Shift Keying (CP-BFSK), wideband FM, and DPSK for UWB communications. The proposed evaluation platform shares the blocks for the spreading factor, bit interleaver, scrambler seed, SRRC filter, low-pass filter, and DDC for both NB and UWB channels, since they have identical configurations.

## 5. Conclusions and Future Work

With the development of modern technology, it becomes possible to establish an intelligent healthcare system that increases the efficiency of conventional medical systems. As the most fundamental element in the intelligent healthcare system, WBAN provides an ultra-low power, reliable, and efficient wireless communication channel for the data exchanging between the sensors and a hub. At the same time, WBAN implementations can be found in other areas, as well, such as that of vehicular networks [10].

However, the lack of an evaluation platform for WBAN systems increases the complexity of designing novel systems for WBAN. Furthermore, evaluating WBAN designs on various platforms could cause unfair performance comparison among different designs intended for the same application. In this paper, an SDR evaluation platform implemented in MiniBEE4 is proposed that supports all the communication configurations specified in the IEEE 802.15.6 standard. To the best of our knowledge, this is the first such reported case of all functioning IEEE 802.15.6 RF channels. Moreover, a demonstration of an NB baseband processing module with the security scheme is set up to verify the performed evaluation platform. The demonstration results proved that the proposed SDR evaluation platform is functional, reliable, and provides the capability to build larger WBAN configurations with more complexity.

In the future, more research attention will be invested in a few additional topics. First, more RF channel cases, such as UWB communication channel evaluation, will be performed exhaustively in the proposed evaluation platform for WBAN systems. Moreover, a more rigorous verification procedure to evaluate WBAN systems in the proposed evaluation platform will be investigated, so that different designs for the same functionality in WBAN, such as the conventional BCH decoder and high-performance BCH decoder, could have fair comparisons.

## Figures and Tables

**Table 1:** Power comparison among different wireless communication technologies [5].

Wireless Communication Technologies	Power (mW)	Battery Life
Bluetooth	5–100	1 day–4 weeks
ZigBee	5–60	3 days–5 weeks
WBAN	0.1–5	6 weeks–12 months

**Table 2:** The central frequency and number of sub-channels in each frequency band.

Frequency Band (MHz)	Relationship between $f_{c}$ and $n_{c}$	Number of Channels
402–405	$f_{c}=402.15+0.30\times n_{c}$ (MHz), $n_{c}=0,\ldots ,9$	10
420–450	$f_{c}=420.30+0.50\times g_{1}\left(n_{c}\right)$ (MHz), $n_{c}=0,\ldots ,11$	12
863–870	$f_{c}=863.20+0.40\times g_{2}\left(n_{c}\right)$ (MHz), $n_{c}=0,\ldots ,13$	14
902–928	$f_{c}=903.20+0.40\times n_{c}$ (MHz), $n_{c}=0,\ldots ,59$	60
950–958	$f_{c}=951.10+0.40\times n_{c}$ (MHz), $n_{c}=0,\ldots ,15$	16
2360–2400	$f_{c}=2361.00+1.00\times n_{c}$ (MHz), $n_{c}=0,\ldots ,38$	39
2400–2483.5	$f_{c}=2402.00+1.00\times n_{c}$ (MHz), $n_{c}=0,\ldots ,78$	79

**Table 3:** Data rate supported on each frequency band.

402–405 MHz	420–450 MHz	863–870 MHz	2360–2400 MHz
		902–928 MHz	2400–2483.5 MHz
		950–958 MHz	
Data Rate (kbps)			
75.9	75.9	101.2	121.4
151.8	151.8	202.4	242.9
303.6	187.5	404.8	485.7
455.4		607.1	971.4

**Table 4:** RF front-end configuration.

FMC111/110	Value
Radio Frequency	1800 MHz
Middle Frequency	30.72 MHz
ADC Sample Rate	250 Msps
Baseband Symbol Rate	31.25 Msps

**Table 5:** Hardware utilization of the baseband processing module performed in the proposed evaluation platform for WBAN systems.

Hardware Resources	Resources Utilized	Utilization Ratio
Lookup tables (LUTs)	14,805/297,600	4%
Registers	11,707/595,200	1%
Memory	81/1064	7%

**Table 6:** Hardware utilization of the security scheme performed in the proposed evaluation platform for WBAN systems.

Hardware Resources	Resources Utilized	Utilization Ratio
LUTs	13,186/297,600	4%
Registers	11,394/595,200	1%
Memory	121/1064	11%
DSPs	190/2016	9%

**Table 7:** UWB operating frequency bands.

Band Group	Channel Number	Central Frequency (MHz)	Bandwidth (MHz)	Channel Attribute
Low band	0	3494.4	499.2	Optional
	1	3993.6	499.2	Mandatory
	2	4492.8	499.2	Optional
High band	3	6489.6	499.2	Optional
	4	6988.8	499.2	Optional
	5	7488.0	499.2	Optional
	6	7987.2	499.2	Mandatory
	7	8486.4	499.2	Optional
	8	8985.6	499.2	Optional
	9	9484.8	499.2	Optional
	10	9984.0	499.2	Optional
